# Facteurs associés à la qualité de la stérilisation du linge opératoire au Centre National Hospitalier Universitaire Hubert Koutoukou MAGA de Cotonou

**DOI:** 10.11604/pamj.2020.35.35.20801

**Published:** 2020-02-10

**Authors:** Comlan Cyriaque Dégbey, Ibrahim Maman Madougou, Charles Sossa, Edgard-Marius Dona Ouendo, Michèl Makoutode

**Affiliations:** 1 Institut Régional de Santé Publique, Université d’Abomey-Calavi, Ouidah, Bénin; 2 Clinique Universitaire d’Hygiène Hospitalière, Centre National Hospitalier Universitaire Hubert Koutoukou MAGA, Cotonou, Bénin

**Keywords:** Stérilisation, infection nosocomiale, linges opératoires, CNHU-HKM, Sterilization, nosocomial infection, surgical drapes, CNHU-HKM

## Abstract

**Introduction:**

La stérilisation des linges opératoires joue un rôle important dans la prévention des infections associées aux soins. Au Centre National Hospitalier Universitaire Hubert Koutoukou MAGA (CNHU-HKM), le processus de stérilisation des linges rencontre des problèmes. La présente étude avait pour objectif d’étudier les facteurs associés à la qualité de stérilisation du linge opératoire au CNHU-HKM.

**Méthodes:**

Il s’agissait d’une étude transversale, descriptive et analytique qui avait porté sur 20 linges opératoires stérilisés, 41 agents impliqués dans la gestion des linges et 55 membres de l’équipe chirurgicale. La méthode avait été probabiliste pour les linges opératoires stérilisés et non probabiliste pour les autres. Le test de Khi carré de Pearson et la régression logistique ont été utilisés pour rechercher l’association avec un seuil significatif et un p <0,05.

**Résultats:**

Quatre-vingt-six virgule quarante-six pourcent des sujets étaient de sexe masculin avec un âge médian de 42 ans. La qualité du processus de stérilisation du linge opératoire n’était pas bonne dans les deux services en charge de traiter les linges. Le contrôle bactériologique avait montré que sur les 20 linges opératoires stérilisés, 9 portaient *Acinetobacter spp.* un germe d’infection nosocomiale multi résistant. L’analyse multivariée avait montré que l’expérience professionnelle (p=0,015) et le contrôle de qualité dans le processus de traitement (p=0,034) sont statistiquement associés à la qualité de la stérilisation.

**Conclusion:**

*Acinetobacter spp.* trouvé sur des linges stérilisés montre que la stérilisation des linges au CNHU-HKM n’est pas de qualité. Un renforcement des compétences des prestataires est nécessaire pour l’amélioration de la qualité du processus de stérilisation.

## Abstract (en)

**Introduction:**

The sterilization of surgical drapes plays an important role in preventing infections associated with treatments. At the CNHU-HKM, sterilization procedure for drapes encounters problems. The purpose of this study was to examine the factors associated with the quality of sterilization of surgical drapes at the CNHU-HKM.

**Methods:**

We conducted a cross-sectional, descriptive and analytical study focusing on 20 sterile surgical drapes, 41 agents were involved in the management of drapes and 55 members of the surgical team. The probabilistic method was used for sterile surgical drapes, the non-probabilistic method for the others. Pearson’s Chi-square Test and logistic regression were used to find the association, with a significant threshold and a p<0.05.

**Results:**

Eighty six point forty six percent of subjects were males with an average age of 42 years. The quality of the process of sterilization of the operative drapes was not good in the two departments responsible for processing the drapes. Bacteriological analysis showed that, out of 20 sterile surgical drapes, 9 had Acinetobacter spp. a multidrug-resistant germ causing nosocomial infections. Multivariate analysis showed that professional experience (p=0.015) and quality control of the procedure (p=0.034) were statistically associated with the quality of sterilization.

**Conclusion:**

The presence of Acinetobacter spp. on the sterilized drapes demonstrates that sterilization of drapes at the CNHU-HKM is of poor quality. Measures strengthening the skills of providers are necessary to improve the quality of sterilization procedures.

## Introduction

Le linge hospitalier en général, est un élément essentiel aussi bien dans les soins que dans le nursing. Il doit assurer un niveau adéquat de sécurité, d’autonomie, d’intimité, d’hygiène et de confort aussi bien pour le malade que pour le personnel soignant. Il participe également à l’image de marque de l’hôpital [1]. Le linge est un élément important dans la lutte contre le risque infectieux. Il joue un rôle de barrière pour protéger patients et soignants d’une contamination par contact ou par des bactéries aéroportées [1, 2, 3]. Sa gestion doit être placée au cœur de la problématique de gestion des infections nosocomiales dans toutes les formations sanitaires. En effet, chaque établissement a son mode de gestion du linge. Avec les restrictions budgétaires, cette fonction a longtemps été considérée sous son seul angle économique. La prise de conscience de la recrudescence des infections nosocomiales, et de leur coût, est en train de faire évoluer de telles attitudes [2]. Le linge opératoire est un cas particulier. C’est un ensemble de dispositif médical réutilisable ou à usage unique, tissé ou non-tissé, composé de tenue de bloc (pantalon et tunique, chaussure, lunette, masque, bonnet), de casaques et champs chirurgicaux utilisé au bloc opératoire lors des interventions chirurgicales. Comme tout linge hospitalier, celui du bloc opératoire joue également le rôle d’effet barrière contre les microorganismes notamment en protégeant d’une part le patient et la plaie opératoire contre les germes provenant de l’environnement et de l’équipe chirurgicale et d’autre part l’équipe chirurgicale contre les germes en provenance du malade (liquide biologique dont le sang avec le risque du VIH, de l’hépatite) [1]. C’est un support de germes. Il joue un rôle important dans la transmission des infections nosocomiales. Sa gestion doit s’intégrer dans une politique globale de prévention contre le risque infectieux. La meilleure manière de gérer le linge opératoire est de l’inscrire dans une démarche qualité d’où la nécessité d’exiger un traitement approprié pour garantir la stérilité de ce dispositif chirurgical. Ce traitement approprié est la stérilisation. C’est une spécialisation de l’activité visant à la prévention des infections nosocomiales. La stérilisation est la destruction de toute forme vivante et en particulier de tous micro-organismes sous formes végétatives ou sporulées, pathogènes ou non [1, 4, 5].

Le Centre National Hospitalier Universitaire-Hubert Koutoukou Maga (CNHU-HKM) de Cotonou dispose de 4 blocs opératoires: Bloc Opératoire Central (BOC), Clinique Universitaire de Gynécologie Obstétrique (CUGO), Clinique Universitaire d’Accueil des Urgences (CUAU) et ORL-Ophtalmologie avec un total de 15 salles opératoires pour une utilisation mensuelle de 1020 tenues de bloc et casaques et de 3487 champs opératoires réutilisables [6]. Le processus de traitement des linges opératoires réalisé au CNHU-HKM ne garantit pas la qualité des linges. La question du linge est très cruciale dans tous les services d’intervention opératoire. Elle s’inscrit dans le cadre d’une action de lutte contre les infections nosocomiales. Une bonne asepsie de la zone opératoire passe par un drapage adapté du patient pour éviter la transmission de germes au niveau du patient. L’équipe chirurgicale a, elle aussi, intérêt à revêtir la tenue la plus sécurisante possible pour bloquer la diffusion de ses propres germes et se protéger de ceux de l’opéré [2]. Le linge hospitalier joue donc un rôle important dans la transmission des infections nosocomiales. Il constitue un enjeu de santé publique. En effet, certains auteurs en ont estimé l’incidence à 17% des infections nosocomiales [2, 7]. Il est donc nécessaire de chercher des voies et moyens pour offrir aux patients et aux soignants un habillement réutilisable de qualité afin de limiter les risques infectieux. En Afrique en général et au Bénin en particulier, peu d’écrits et d’études scientifiques sont réalisés sur la qualité du système de stérilisation des linges opératoires. C’est dans ce contexte que s’inscrit la présente étude, qui vise la recherche des facteurs associés à la qualité de la stérilisation du linge opératoire au CNHU-HKM de Cotonou.

## Méthodes

Ce travail a été réalisé au CNHU-HKM de Cotonou. Il s’agissait d’une étude transversale, descriptive et analytique. La population d’étude était composée de cibles primaires et secondaires. Les cibles primaires étaient constituées des linges opératoires des blocs de BOC, CUAU, CUGO et ORL-Ophtalmologie et des agents impliqués dans le processus de gestion des linges du CNHU-HKM à savoir les opérateurs de la buanderie et de la stérilisation ainsi que les collecteurs des quatre blocs opératoires. Quant aux cibles secondaires, elles étaient constituées de: utilisateurs des linges opératoires (équipe chirurgicale); Chef Service de l’Economat; Chef Service de la maintenance; Chef de Division Section Auxiliaire; Chef de Division Formation du personnel; Surveillante du Service Hygiène Hospitalière; Chef de Section Buanderie; Responsable stérilisation et le Président du CLIN. La méthode et la technique d’échantillonnage avaient été déterminées par cible. Concernant les blocs opératoires, la méthode utilisée était non probabiliste avec comme technique le choix raisonné. Ce choix reposait d’une part sur le nombre d’intervention chirurgicale par jour et la fréquence d’envoi des linges sales et propres respectivement à la buanderie et à la stérilisation. D’autre part sur le nombre journalier de tambour a préparé. Pour le choix des tambours stérilisés qui contenaient les linges opératoires stériles, la méthode utilisée était non probabiliste avec un choix raisonné. Quant aux linges stérilisés, la méthode utilisée était probabiliste et nous avons procédés au choix aléatoire simple pour effectuer le prélèvement.

Le prélèvement du linge opératoire stérile avait consisté à frotter 2 fois à l’aide de deux différents écouvillons stériles sur un seul et même linge stérile contenu dans un tambour stérile juste quelques secondes après son ouverture dans la salle opératoire et avant l’habillement du chirurgien. Le prélèvement ainsi effectué est placé dans un tube stérile contenant de l’eau distillé pour être analysé au laboratoire. L’analyse des données a consisté en des analyses statistiques et des analyses en laboratoire. Le test de Khi carré de Pearson et la régression logistique ont été utilisés pour rechercher l’association avec un seuil significatif et un p <0,05. Pour l’analyse en laboratoire, chaque échantillon avait fait l’objet aussi bien systématiquement d’un examen macroscopique, microscopique composé de l’état frais, de la coloration de Gram puis d’un ensemencement sur milieux ordinaire et sélectif en vue d’apprécier les comportements des bactéries pour la suite des travaux. Les différents milieux de culture utilisés étaient: (bouillon Mueller Hinton (MH), gélose eosin methylene blue (EMB), gélose CHAPMAN, gélose au sang ordinaire et avec acide nalidixique colistine (ANC), gélose chocolat avec poly vitex). Pour les tests d’identification, dans notre étude nous n’avions eu que des bacilles gram négatifs. L’identification des bactéries a été faite sur la galerie API 20E. Le test de sensibilité avait été effectué en même temps que le test d’identification. La méthode utilisée est celle de Kirby Bauer ou par diffusion des disques imprégnés d’antibiotiques sur milieu solide. La gélose a été ensuite incubée à 37°C pendant 24 heures à l’étuve. Par ailleurs, la concentration minimale inhibitrice (CMI) a été faite et comparée à l’étalon de Mac.

## Résultats

**Caractéristiques de la population d’étude:** notre étude avait porté d’une sur 96 agents du CNHU-HKM dont 86,46% étaient de sexe masculin avec un âge médian de 42 ans. D’autre part sur 20 linges opératoires en provenance de BOC, CUAU et CUGO.

### Description du processus de gestion des linges opératoire au CNHU-HKM de Cotonou

**Processus de gestion de linge à la buanderie du CNHU-HKM:** la section Buanderie, Lingerie et Taillerie du CNHU-HKM a pour mission d’assurer la confection, l’entretien et la propreté des divers linges hospitaliers à savoir les blouses, les casaques, les champs opératoires, les bottes etc. Pour accomplir cette mission, la buanderie dispose de neuf (9) agents dont 5 permanents (conventionnés) et 4 contractuels (occasionnels). Durant notre étude, ce sont les quatre occasionnels qui s’occupaient permanemment du lavage des linges. Le lavage des linges sales à la buanderie du CNHU-HKM se fait tous les jours à partir de 6 heures du matin, heure locale. Il est assuré par deux machines automatisées de lavage qui disposent d’un système de désinfection, d’aromatisation et d’essorage incorporé. Le processus du lavage se fait suivant les étapes suivantes: stockage: très tôt, les agents de garde des différents blocs ou services déposent les linges sales dans la zone sale; tri et séparation: il est assuré par les laveurs et consiste à séparer les draps, les linges opératoires et les blouses du personnel de chaque service; vérification d’objet: elle consiste à vérifier d’une part la présence d’instruments opératoires dans les linges qui sont susceptibles de nuire au bon fonctionnement des appareils et d’autre part la formation des caillots de sang dû au temps d’envoi des linges sales; dosage des produits: le dosage des produits lessiviels se fait dans les 4 bacs que disposent les appareils: un bac pour le détergent, deux bacs pour le désinfectant et un bac pour les produits additifs (aromatiques); chargement, lancement et principe de fonctionnement des appareils: après avoir chargé les cuves et lancé le processus, les machines mettent 1 heure de temps pour une température à chaud de 90°C. Dans un premier temps, elles utilisent les détergents pour une phase de lavage et de 1^er^ rinçage. Ensuite une seconde phase de lavage-désinfection avec utilisation du 1^er^ bac à désinfectant et terminée par un 2^ème^ rinçage. Une 3^ème^ phase de lavage-désinfection avec utilisation du 2^ème^ bac à désinfectant et achevée par un 3^ème^ rinçage. Enfin, une phase d’essorage à laquelle les machines annoncent la fin du processus; déchargement: le déchargement se fait manuellement par les opérateurs et transporter dans des chariots vers les zones de séchage. Spécialement, le bloc CUAU préfère sécher ses linges propres dans les enceintes du service des urgences; séchage: il est assuré par les opérateurs de la buanderie en ce qui concerne les linges opératoires de BOC et de CUGO.

**Processus de gestion de linge dans les blocs opératoires: préparation des tambours:** elle s’effectue dans les quatre (4) blocs opératoires par les aides-soignants qui sont en même temps les agents collecteurs. Après avoir récupérés les linges propres à la buanderie, ces agents collecteurs procèdent à la vérification des taches sur les linges propres, leur pliage et ensuite leur conditionnement dans des tambours en inox ou en aluminium pour être acheminé vers la stérilisation du bloc opératoire central.

**Processus de gestion de linge à la stérilisation du bloc opératoire:** elle a pour mission d’assurer le bon fonctionnement des blocs opératoires de l’hôpital en leur fournissant du matériel stérile. Elle dessert principalement le bloc opératoire central de l’hôpital et les blocs de CUGO, CUAU, OPHTALMO-ORL, etc. On constate que les murs sont rugueux, non lavés avec des corniches qui retiennent la poussière; les dessous de lavabos sont ouverts avec des tuyaux de canalisation couverts de poussières; les surfaces (tables) sont en bois non couvertes et servent pour la préparation des boîtes de matériels, le repli du linge et des compresses à stériliser. L’accès à l’unité est permis à tout usager venant de l’extérieur y compris les vendeurs ambulants, le vestiaire n’a pas d’issue sur l’extérieur, le personnel est obligé de traverser la zone propre avant d’y accéder pour se changer et porter la tenue professionnelle. Ainsi, le principe de la marche en avant, plus ou moins respecté pour le matériel médico-technique, ne l’est pas pour le personnel du service. La stérilisation du linge opératoire est assuré par une autoclave de marque subtil crepieux d’une capacité de 500 litres. Elle comprend les phases suivantes: la phase avant stérilisation: la vérification des tambours et addition des rubans indicateurs, la vérification du fonctionnement de l’appareil, le chargement de l’appareil et le préchauffage; la phase de stérilisation: après le choix et lancement du cycle, l’autoclave du CNHU-HKM suit les étapes suivantes pour stériliser le matériel (tambours autoclavables contenant les linges opératoires): phase initiale, 6 phases d’injection, 6 phases de détente, un vide intermédiaire, le chauffage, le plateau (stérilisation proprement dite), l’évacuation, le vide final, le balayage, mise à l’atmosphère, l’impression. A la fin du cycle, l’autoclave annonce que le cycle est terminé et qu’on peut ouvrir la porte; la phase après stérilisation: elle est essentiellement caractérisée par le déchargement des tambours, la vérification du virage des indicateurs physico-chimiques et la siccité des tambours.

**Qualité physico-chimique du traitement de linge opératoire à la buanderie:** la mesure de la qualité physico-chimique de lavage a consisté à l’observation de trois (3) cycles de lavage-stérilisation de linges en provenance de trois (3) blocs opératoires. A la buanderie, nous avons constaté que sur les trois (3) cycles des trois (3) blocs opératoires, l’absence de contrôle de routine des paramètres physiques de lavage, l’utilisation de produits lessiviels de mauvaise qualité, le non-respect du dosage des produits lessiviels, le non utilisation des produits aromatiques, l’absence de supervision des agents au travail, le non-respect de la quantité de linges à laver, l’absence de rinçages supplémentaires, l’absence de contrôle visuel de la qualité de l’eau de rinçage, l’absence de contrôle de la qualité de l’eau et de l’air. A l’unité de stérilisation, nous avons constaté l’absence de test d’homogénéité au vide, l’absence de test de Bowie et Dick, l’absence de contrôle technique (contrôle routinier des paramètres physiques de stérilisation, du diagramme de stérilisation, vérification du virage des intégrateurs).

**Qualité microbiologique du linge opératoire:** la qualité du processus de stérilisation du linge opératoire n’était pas bonne dans les deux services en charge de traiter les linges. Le contrôle bactériologique a montré que sur les 20 linges opératoires stérilisés, 9 portaient *Acinetobacter spp.* un germe d’infection nosocomiale multi résistant (Tableau 1).

**Tableau 1:** Qualité microbiologique du linge opératoire prélevé au niveau des blocs du CNHU-HKM

Bloc opératoire	Tambour de prélèvement	Linge opératoire	Culture	Germes identifiés
BOC	N°1	Casaque	-	
	N°2	Casaque	-	
	N°3	Casaque	-	
	N°4	Champs opératoire	-	
	N°5	**Champs opératoire**	+	***Acinetobacter sp.***
	N°6	Champs opératoire	-	
	N°7	Casaque	-	
	N°8	Champs opératoire	-	
	N°9	**Essuie-main**	+	***Acinetobacter sp.***
	N°10	**Essuie-main**	+	***Acinetobacter sp.***
CUGO	N°1	Casaque	-	
	N°2	Casaque	-	
	N°3	**Casaque**	**+**	***Acinetobacter sp.***
	N°4	**Champs de table**	**+**	***Acinetobacter sp.***
	N°5	**Blouse**	**+**	***Acinetobacter sp.***
CUAU	N°1	Champs opératoire	-	
	N°4	Blouse	-	
	N°2	**Casaque**	**+**	***Acinetobacter sp.***
	N°3	**Casaque**	**+**	***Acinetobacter sp.***
	N°5	**Champs opératoire**	**+**	***Acinetobacter sp.***

**BOC**: Bloc Opératoire Central

**CUGO**: Clinique Universitaire de Gynécologie Obstétrique

**CUAU**: Clinique Universitaire d’Accueil des Urgences

### Facteurs associés à la qualité de stérilisation du linge opératoire du CNHU-HKM

**Facteurs liés au personnel impliqué dans le processus de gestion des linges:** parmi les facteurs liés au personnel impliqué, seuls l’expérience professionnelle (p=0,049) et le statut social (p=0,009) sont statistiquement associés à la qualité de la stérilisation du linge opératoire.

**Facteurs liés aux services chargés de traiter les linges:** il existait une association statistiquement significative entre la qualité de la stérilisation du linge opératoire et les facteurs techniques (approvisionnement en intrant, disponibilité de matériels de travail, traçabilité) liés au système de gestion des linges (Tableau 2).

**Tableau 2:** Association entre la qualité de la stérilisation du linge opératoire et les facteurs liés aux services chargés de traiter les linges (n=96)

Facteurs liés au personnel impliqué	Qualité de la stérilisation du linge			P-value
	Oui	Non	Total	
Approvisionnement en Intrant				
Oui	1	9	10	0,016*
Non	43	43	86	
Disponibilité de matériels de travail				
Oui	4	9	13	0,015*
Non	40	43	83	
Traçabilité				
Oui	12	40	52	0,029*
Non	3	41	44	

* **Significatif**

**Facteurs liés au système de gestion des linges:** l’analyse du Tableau 3 nous montre qu’il y a une association statistiquement significative entre la qualité de la stérilisation du linge opératoire et les facteurs tels que le bon fonctionnement des appareils de traitement, la maintenance préventive et le contrôle technique.

**Tableau 3:** Association entre la qualité de la stérilisation du linge opératoire et les facteurs techniques liés au système de gestion des linges

Facteurs liés au personnel impliqué	Qualité de la stérilisation du linge			P-value
	Oui	Non	Total	
**Aération des zones de traitement de linge**				0,050
Oui	2	43	52	
Non	42	42	44	
**Exigüité des zones de traitement de linge**				0,077
Oui	3	10	13	
Non	41	42	83	
**Bon fonctionnement des appareils de traitement**				0,018*
Oui	2	11	13	
Non	42	41	83	
**Dispositif de l’hygiène des mains**				
Oui	2	5	52	0,341
Non	42	47	44	
**Lumière**				0,329
Oui	15	13	28	
Non	29	39	68	
**Maintenance corrective**				0,757
Oui	5	7	12	
Non	39	45	84	
**Maintenance préventive**				0,030*
Oui	2	10	12	
Non	42	42	84	
**Contrôle technique**				
Oui	2	11	13	0,018*
Non	42	41	83	
**Protocole de traitement**				0,110
Oui	6	14	10	
Non	38	38	76	

**Analyse multivariée:** l’analyse multivariée par régression logistique a montré que l’expérience professionnelle (p=0,015) et le contrôle de qualité dans le processus de traitement (p=0,034) sont statistiquement associés à la qualité de la stérilisation (Tableau 4). L’adéquation du modèle a été effectué à partir du test de Hosmer-Lemeshow; on obtient p=0,5403>5%. Le test n’étant pas significatif, on conclut la bonne adéquation du modèle logistique. Finalement les variables: expérience professionnelle et contrôle technique sont effectivement associées à la qualité de stérilisation du linge opératoire. L’expérience professionnelle étant un facteur de protection à la qualité de stérilisation du linge opératoire au CNHU de Cotonou.

**Tableau 4:** Facteurs associés à la qualité de la stérilisation du linge opératoire au CNHU-HKM

Variable explicative	Effectif total	Mauvaise qualité		OR ajusté	IC (95%)	P-value
		Effectif	%			
**Expérience professionnelle**						**0,015**
< 6ans	44	22	50	1		
> 6ans	52	30	57,7	0,34	[0,14; 0,81]	
**Contrôle technique**						**0,034**
Oui	83	41	49,39	1		
Non	13	11	84,61	5,62	[1,14; 27,82]	

## Discussion

La recherche des facteurs associés à la qualité de stérilisation du linge opératoire au CNHU-HKM de Cotonou a été réalisée à partir d’une étude transversale, descriptive et analytique, basée sur l’exploitation documentaire, l’enquête par questionnaire, l’observation, l’entretien et les prélèvements effectués sur des linges stérilisés. Il s’agit de la première étude réalisée au Bénin sur cette thématique. Les données scientifiques qui la concernent sont très rares.

### Description du processus de gestion des linges opératoire au CNHU-HKM de Cotonou

**Processus de gestion de linge à la buanderie du CNHU-HKM:** l’observation de la zone de traitement de linge de la buanderie du CNHU-HKM nous a permis de constater les conditions de travail dans lesquelles travaillaient les agents. En effet, l’encombrement du local, le manque de dispositif de l’hygiène des mains, l’inobservance du bionettoyage ainsi que l’insuffisance d’aération et de lumière dans cette zone sont autant des facteurs qui ne garantissent pas le confort aux opérateurs sur le lieu de travail. Ce qui pourrait affecter la qualité du lavage. Ces observations sont similaires à celles constatées par Ayelo AP *et al.* [8] à la buanderie de l’Hôpital de la Mère et de l’Enfant Lagune de Cotonou en 2013. Au CNHU-HKM, le lavage des linges est assuré à 66,66% par des agents occasionnels non qualifiés et sans aucune expérience professionnelle. Cette situation ne garantit pas la qualité du traitement. Le processus de traitement de linge à la buanderie du CNHU souffre de beaucoup de d’anomalies. En effet, l’observation des agents à la tache nous a permis de constater le non-respect des étapes du processus de lavage notamment le séchage où 3cas/3 des essuies mains propres de CUGO ont été étalés au sol pour être séchés. Cela peut s’expliquer par le fait que ces agents n’ont pas la notion de risque infectieux hospitalier. Or, par principe on ne stérilise que ce qui est propre. Les chariots de transport des linges propres vers les lieux de séchage après chaque cycle de lavage ne sont pas laver. Cette pratique est contraire aux recommandations du Conseil Supérieur d’Hygiène de Belgique en matière de traitement de linge [9]. La gestion des linges opératoires à la buanderie ne fait pas l’objet de traçabilité malgré son importance dans la protection juridique des opérateurs et de l’institution. Cela peut s’expliquer par le manque d’un service de qualité des soins au CNHU qui doit surveiller cette activité car elle constitue une part essentielle d’un système de qualité. Aussi la préparation des tambours qui devraient se faire à la lingerie de la buanderie se fait dans les services opératoires. Ce comportement est contraire aux bonnes pratiques de gestion des linges. Il se justifie par le fait que les tous services opératoires n’ont pas confiance à la lingerie du CNHU. Au cours de notre étude, nous avons constaté que la quantité de linge par tambour autoclavable n’est pas respectée par tous les services opératoires notamment par le bloc opératoire central. C’est pour éviter une rupture en linge stérilisé du fait des problèmes que rencontrent l’unique autoclave qui dessert tous les blocs opératoires du CNHU.

**Processus de gestion de linge à la stérilisation du CNHU-HKM:** le Centre National Hospitalier et Universitaire Hubert Koutoukou Maga de Cotonou ne dispose pas de service de stérilisation centrale. Tous les quatre services Bloc Opératoire Central (BOC), Clinique Universitaire d’Accueil des Urgences (CUAU), Clinique Universitaire de Gynécologie Obstétrique (CUGO) et Oto-Rhino-Laryngologiste (ORL) qui ont fait l’objet de notre étude disposent des stérilisateurs. Cependant, en dehors de celui du BOC ils sont non fonctionnels. C’est donc l’unité de stérilisation de BOC qui est l’autorité responsable de la stérilisation des linges opératoires au CNHU. La technique utilisée est celle de la chaleur humide conformément à la recommandation de l’OMS. La stérilisation par chaleur humide (autoclave) garantit la plus grande sécurité [10]. L’agent stérilisant est la vapeur d’eau saturée qui permet la dénaturation des protéines bactériennes par hydrolyse [11]. A l’unité de stérilisation de BOC du CNHU, nous avons constaté l’inadéquation architecturale de son local et la vétusté de ces appareils dont l’unique qui assurait le travail est en perpétuel dysfonctionnement.

Ces observations sont identiques à celles observées par Ouendo EM *et al.* [12] en 2013 dans le même hôpital. Deux ans après les mêmes constats se répètent dans le même service. Ceci traduit le manque de volonté des autorités hospitalières à prendre en considération les problèmes de stérilisation au CNHU, qui est pourtant un maillon essentiel de la qualité des soins et service au sein d’un l’hôpital puisqu’elle joue un rôle important dans la lutte contre les infections associées aux soins. Aussi, nous avons constaté l’absence notoire de contrôle technique en stérilisation pour s’assurer de la qualité du procédé mis en œuvre. Cela peut s’expliquer par le fait que les agents de stérilisation n’ont pas la notion de démarche qualité. Alors que cette pratique est une exigence dans les pays européens [13]. Au CNHU-HKM, les 3 agents de garde qui assuraient les 70% du travail de stérilisation sont des aides-soignants. Ils n’ont jamais été supervisés ni évalués depuis leur prise de fonction, aucun d’entre eux n’a eu l’occasion de suivre une formation continue en stérilisation, malgré l’évolution rapide de la science et de la technologie. Les mêmes constats ont étés observés en 2013 par Ouendo EM *et al.* [12] dans son étude. Cet état de chose influencerait non seulement la qualité du matériel mis à la disposition des praticiens mais aussi la qualité des soins et services offerts aux patients dans le plus grand hôpital du pays [8]. Le processus de stérilisation des linges opératoires tel que ça se passe au CNHU ne saurait garantir la qualité. En effet, les multiples disfonctionnements de l’unique autoclave, qui peuvent s’expliquer par une fuite de responsabilité de la maintenance biomédicale et le non-respect des étapes de stérilisation par les opérateurs sont autant de facteurs qui ne garantissent pas la qualité de stérilisation au CNHU. A cela s’ajoute l’inexistence des protocoles de désinfection et de stérilisation au niveau de l’hôpital comme l’a aussi constaté Ayelo AP *et al.* [8] en 2013 dans leur observation. Selon ces auteurs, cette absence de protocole pourrait s’expliquer non seulement par l’inobservance de la démarche qualité, aujourd’hui incontournable dans tout établissement hospitalier mais aussi par l’inexistence des équipes d’expert ayant travaillé dans le domaine.

**Qualité physico-chimique du linge opératoire stérilisé:** la qualité physico-chimique du linge opératoire stérilisé découle de la qualité de son traitement dans les deux services responsables du traitement de linges opératoire au CNHU à savoir la buanderie et la stérilisation. La mesure de la qualité physico-chimique de lavage avait consisté à l’observation de 3 cycles de lavage-stérilisation de linges en provenance de 3 blocs opératoires. Le dosage des produits lessiviels ainsi que la quantité de linge à laver par cycle de lavage sont des déterminants essentiels du cycle de lavage. A la buanderie du CNHU, ils ne sont pas respectés. Ce qui pourrait avoir des conséquences sur la qualité de lavage. Cette situation pourrait s’expliquer d’une part par le fait que le service ne dispose pas d’instrument de mesure des produits lessiviels et de pesée pour les linges et d’autre part l’inexistence de protocole de lavage et l’absence de supervision des opérateurs. La qualité de lavage est aussi fonction de la qualité des produits lessiviels. Nous avons constaté que la buanderie utilisait comme détergent «OMO super active» et comme désinfectant une solution chlorée fabriquée à la pharmacie. Ces produits lessiviels ne sont pas de qualité et ne permettent pas d’obtenir de linge de qualité. Cette situation pourrait être due à l’insuffisance de la politique d’approvisionnement de la buanderie.

L’absence de contrôle technique (contrôle visuel de la qualité de l’eau de rinçage, contrôle de la qualité de l’air et de l’eau, contrôle de routine des paramètres physiques de lavage notamment la température et la durée des phases et du cycle pourraient s’expliquer d’une part par le fait que les agents buandiers ne sont pas formés en technique de lavage des linges hospitaliers et d’autre part, l’encombrement et l’insalubrité du local sont des facteurs qui ne favorisent pas la réalisation de cette activité. A l’unité de stérilisation, nous avons constaté l’absence de test d’homogénéité au vide, l’absence de test de Bowie et Dick et l’absence de contrôle technique (contrôle routinier des paramètres physiques de stérilisation, du diagramme de stérilisation, vérification du virage des intégrateurs). Ces défauts ne permettent pas d’offrir un linge opératoire stérilisé de qualité aux patients et à l’équipe chirurgicale et sont contraires aux bonnes pratiques de stérilisation en se référant à l’Association Française de Stérilisation et la Société Suisse de Stérilisation Hospitalière qui évoluent dans une logique d’optimisation de la qualité comme une démarche permanente pour offrir la garantie d’une qualité de soins optimale aux patients [14]. Ces défauts pourraient s’expliquer d’une part par l’insuffisance de la politique d’approvisionnement en intrant chimique et d’autre part le manque de formation des agents stérilisateurs et aussi l’insuffisance dans la maintenance de l’autoclave.

**Qualité bactériologique du linge opératoire stérilisé:** l’analyse bactériologique effectuée après stérilisation au laboratoire de CNHU a permis d’identifier *Acinetobacter spp.* sur 9 cas/20 de linges prélevés après stérilisation. Ce sont des germes de l’infection nosocomiale, multi résistants aux antibiotiques, qui se traduisent dans les septicémies. En France en décembre 2003, *Acinetobacter baumannii* était responsable de 112 infections et 18 décès dans les milieux hospitaliers du nord [15]. Nos résultats sont similaires à ceux de Dégbey C *et al.* en 2013 [10] dans la même unité de stérilisation. Contrairement à notre étude, ces auteurs avaient travaillé sur les matériels médico-techniques utilisés dans les blocs opératoires et ont identifié les germes suivant: *Staphylococcus saprophyticus, Staphylococcus aureus, Enterobacter agglomerans,* bacilles à gram négatif et *Pseudomonas aruginosa.* Ils avaient incriminé la contamination de l’air par le nombre élevé de personnes présentes en salle d’intervention, la défaillance dans la chaîne de stérilisation et de désinfection des dispositifs médicaux utilisés, la mauvaise conception du BOC opératoire et la défaillance dans la désinfection des surfaces et locaux et de l’entretien. L’identification de *Acinetobacter spp.* sur les linges opératoires stérilisés du CNHU dans notre étude pourrait s’expliquer d’une part par les germes manuportés lors du transport et le stockage des tambours de la stérilisation vers les services du fait de l’inobservance de l’hygiène des mains des manipulateurs; la qualité du linge opératoire (vétuste) et sa capacité de perméabilité aux germes et d’autre part par l’absence de contrôle biologique de l’autoclave du CNHU. Ce contrôle biologique consiste à mettre des tests biologiques dans une charge dans les mêmes conditions d’emballage que le matériel à stériliser. La stérilisation d’un dispositif médical signifie l’obtention d’un germe zéro sur ce dispositif. *Acinetobacter spp.* germe multirésistant obtenu sur 9cas/20 de linges opératoires après leur stérilisation témoigne de la mauvaise qualité du processus de stérilisation du linge opératoire au CNHU-HKM.

**Facteurs associées à la qualité de stérilisation du linge opératoire:** le statut social plus précisément l’agent occasionnel est associé à la qualité de stérilisation du linge opératoire (p=0,020 avec OR=14). Cette association peut s’expliquer par le fait que ces agents, auxquels on confie le traitement des linges sont sans aucune formation de base en traitement des linges. Ils sont recrutés de façon saisonnière par le CNHU et bénéficient d’un contrat de trois mois renouvelable une seule fois. Parmi cette catégorie se trouvent les aides-soignants. Ils sont au bas de l’échelle de la pyramide sanitaire, n’ont pas été formés dans une école professionnelle spécialisée, oubliés dans les formations continues, moins écoutés et appelés à exécuter les tâches les plus «dures» [10]. Cet état de chose influencerait non seulement la qualité du linge opératoire mis à la disposition des praticiens mais aussi la qualité des soins et services offerts aux patients. L’expérience professionnelle (p=0,019 et OR<1) est aussi associée à la qualité de la stérilisation du linge opératoire. Le résultat obtenu signifie que le risque de donner un linge stérilisé de mauvaise qualité dimunie significativement avec l’expérience professionnelle. C’est donc un facteur protecteur pour la qualité de stérilisation du linge opératoire. Or le personnel qui s’occupe du traitement des linges au CNHU est un personnel jeune et sans expérience professionnelle. Cet état de fait sans doute influencerait la qualité du traitement. S’agissant des facteurs liés aux services chargés de traiter les linges, l’approvisionnement en intrant chimique (p=0,016 et OR ajusté=9) est fortement associé à la qualité de stérilisation du linge opératoire. Cela signifie que l’approvisionnement en intrant chimique de qualité des services chargés de traiter les linges déterminerait sans doute la qualité de stérilisation des linges opératoires.

Au CNHU, nous avons constaté que la dotation en intrant chimique de qualité à la buanderie et à la stérilisation n’est pas garantie. Dans ces conditions, la qualité de la prestation ne sera meilleure. La disponibilité des matériels (p=0,015 et OR ajusté=4,05) est significativement associée à la qualité de stérilisation de linge. Ce résultat traduit que l’obtention d’un linge opératoire bien stérilisé dépend de la dotation des services de buanderie et de stérilisation en matériels adéquats. La traçabilité du traitement de linges (p=0,0029 et OR ajusté=4,1) est associée à la qualité de la stérilisation. Cela peut s’expliquer par le fait que la traçabilité est un élément important de la démarche qualité. Au CNHU, la traçabilité existe dans les services chargés de traiter les linges. Mais elle est insuffisante et mérite d’être corrigée pour qu’elle joue pleinement son rôle dans la gestion des linges. En ce qui concerne les facteurs techniques liés au système de gestion, trois facteurs ont été associés à la qualité de stérilisation du linge opératoire. Il s’agit du bon fonctionnement des machines de traitement, de la maintenance préventive et du contrôle technique du processus de traitement. La probabilité d’obtenir des linges opératoires qualitativement stériles à la stérilisation du CNHU dépend d’une part du bon fonctionnement et de l’entretien des machines et de l’autre part de la qualité des contrôles techniques avant, pendant et après chaque processus de traitement. L’analyse multivariée nous a permis de retenir deux facteurs qui ont été effectivement associés à la qualité de stérilisation du linge opératoire. Il s’agit de l’expérience professionnelle et du contrôle technique du processus de traitement. L’explication de leur association réside du fait que le contrôle technique du processus de traitement des linges est le bras armé de la qualité de stérilisation. L’expérience professionnelle est un facteur de protection à la qualité de traitement.

## Conclusion

Le processus de gestion du linge opératoire au CNHU-HKML montre des insuffisances qu’il est nécessaire de corriger la présence d’un germe d’infection nosocomiale *(Acinetobacter spp.)* multi résistant sur des linges opératoires après stérilisation attestent la mauvaise qualité du processus de stérilisation du linge opératoire au CNHU-HKM de Cotonou. La recherche d’association à la qualité de stérilisation du linge opératoire au CNHU a permis de retenir deux facteurs qui sont réellement incriminés. Il s’agit de l’expérience professionnelle et le contrôle technique du processus de traitement des linges. Pour résoudre ce problème, des actions doivent être entreprises afin d’atteindre un niveau de stérilisation de qualité acceptable.

### Etat des connaissances actuelles sur le sujet

- La stérilisation des linges opératoires joue un rôle important dans la prévention des infections associées aux soins;
- Comme tout linge hospitalier, celui du bloc opératoire joue le rôle d’effet barrière contre les microorganismes notamment en protégeant d’une part le patient et la plaie opératoire contre les germes provenant de l’environnement et de l’équipe chirurgicale et d’autre part l’équipe chirurgicale contre les germes en provenance du malade (liquide biologique dont le sang avec le risque du VIH, de l’hépatite;
- Peu d’écrits et d’études scientifiques sont réalisés sur la qualité du système de stérilisation des linges opératoires.

### Contribution de notre étude à la connaissance

- Cette étude montre la défaillance de la qualité du processus de stérilisation des linges opératoires;
- La recherche d’association à la qualité de stérilisation du linge opératoire au CNHU-HKM a permis de retenir deux facteurs qui sont réellement incriminés. Il s’agit de l’expérience professionnelle et le contrôle technique du processus de traitement des linges;
- L’étude met en évidence la nécessité de renforcer les compétences des prestataires pour l’amélioration continue de la qualité du processus de stérilisation.

## Remerciements

Les auteurs remercient tous les professionnels de la buanderie et de l’unité de stérilisation du CNHU-HKM pour leur contribution à la réalisation de cette étude.
